# Physicians’ Mutual Benefit Association

**Published:** 1888-02

**Authors:** 


### THE PHYSICIANS' MUTUAL BENEFIT ASSOCIATION OF TEXAS.

It is proposed during the stay in Galveston, of members visiting the State Association Convention, to hold a meeting of the Physicians' Mutual Benefit Association.

It is necessary that there should be a meeting. There are to be elected a board of directors; and the Secretary wishes to submit a report. There are also certain changes in the Constitution and By-Laws to be proposed for the better working of the body. It was the intention to publish in this issue several letters from members, conveying suggestions for the improvement of our operations, together with the Constitution and By-Laws; but, in consequence of one or two long articles of interest, it is not possible to do so. In the next issue, however, it is proposed to publish these letters, and the Constitution and By-Laws, together with an appeal to the profession to take an interest in the matter. In the next issue will be published, also, the names of all who paid the annual dues, and all who shall have paid the assessment No. 5. (Dr. Starley). The 30 days have expired, and though the large majority have paid, we regret to say there are still a great many who have not done so, and second notices have been sent them. We think best to do this, and to give all a thorough chance to keep their pledge—rather than to promptly suspend them at the end of 30 days; because, in the case of assessment No. 4, and of annual dues, it was found that many did not receive the first notice—some, even the second notice, and finally several paid as late as 60 days after date of first notice. We had hoped to be able to forward the entire amount of assessment ere now, and to publish the names of members paying, together with Mrs. Starley's receipt; but the latter will have to be postponed 'till next issue; meantime, the net result of the assessment will be forwarded as soon as all have paid who intend to pay. It is a source of much regret that we are compelled to report the forfeiture ot quite a number ot members for non-payment of the annual dues; hence, assessment No. 5 will not realize as much money as assessment No. 4

In our last we published the results of the preceding four assessments. If, with an average membership of about 110 for four years, it is possible to present, as a free-will offering to the widows of four physicians—an average of $106 each, what could be done with a membership of 1000, and an assessment of $2.50 instead of $1.00? If, with a membership of 100the loss has averaged one death a year, with 1000 the loss would likely be ten a year. With an assessment of $2.50 this would cost $25 a year to safely carry an insurance of $2,500, which is fifty per cent less than any insurance extant. Think of it!

It is desired to get up to these figures so as to make the insurance not only an object, but a real benefit to a deceased brothers' family. One leading physician of the State—a man of large benevolence and a heart full of charity and sympathy, *resigned* his membership under, doubtless, a misapprehension of facts. He said he had all the insurance he needed (or wanted). So have many, doubtless, but this organization is not for those fortunate ones who are able to provide insurance, as well as all the comforts of life, for their families, but for those who, having devoted their lives to the care of others, find themselves in their older age either *too old or too poor* to secure life insurance, or are physically unable to stand the required examination; to the widows of such, even $100 is *acceptable*, to say the least; but suppose it were possible to secure to them $2500? This can just as easily be done, if the profession of Texas will take hold of the *nucleus* which, for four years, has been struggling for life.

